# Assessment of CFTR Dysfunction and Responsiveness to CFTR Modulators in COPD Bronchial Air–Liquid Interface Cultures

**DOI:** 10.3390/ijms27198803

**Published:** 2026-10-01

**Authors:** Nikhil T. Awatade, Kurtis F. Budden, Prabuddha S. Pathinayake, Andrew T. Reid, Kristy S. Nichol, Peter A. B. Wark

**Affiliations:** 1 Immune Health Program, Hunter Medical Research Institute, School of Medicine and Public Health, University of Newcastle, Newcastle, NSW 2308, Australia; kristy.nichol@newcastle.edu.au; 2 School of Medicine and Public Health, University of Newcastle, Newcastle, NSW 2308, Australia; prabuddha.pathinayake@newcastle.edu.au (P.S.P.); andrew.reid@newcastle.edu.au (A.T.R.); 3 Impact and Evaluation, Hunter Medical Research Institute, Locked Bag 1000, New Lambton Heights, Newcastle, NSW 2305, Australia; kurtis.budden@newcastle.edu.au; 4 School of Biomedical Sciences and Pharmacy, College of Health, Medicine and Wellbeing, University of Newcastle, Newcastle, NSW 2308, Australia; 5 Infection Research Program, Hunter Medical Research Institute, School of Biomedical Sciences and Pharmacy, University of Newcastle, Newcastle, NSW 2308, Australia; 6 Breathing and Lung Health Research Program, Hunter Medical Research Institute, New Lambton Heights, Newcastle, NSW 2305, Australia; 7 Department of Respiratory and Sleep Medicine, John Hunter Hospital, Newcastle, NSW 2305, Australia; 8 School of Translational Medicine, Monash University, Melbourne, VIC 3800, Australia; 9 Respiratory Medicine, Alfred Hospital, Melbourne, VIC 3004, Australia

**Keywords:** airway epithelium, COPD, CFTR, air–liquid interface, icenticaftor, ivacaftor

## Abstract

Increasing evidence suggests that acquired dysfunction of the cystic fibrosis transmembrane conductance regulator (CFTR) anion channel occurs as a result of cigarette smoke exposure in chronic obstructive pulmonary disease (COPD). CFTR-targeted therapies were developed to treat genetic CFTR defects in cystic fibrosis, but they have not demonstrated consistent clinical efficacy in small trials of patients with COPD. Here, we aimed to characterize CFTR and other ion channel activity and ciliation in primary bronchial epithelial cells (pBECs) from COPD donors compared with healthy controls, and to determine the extent to which CFTR dysfunction can be rescued by clinically relevant CFTR modulators. Air–liquid interface (ALI) cultures generated from conditionally reprogrammed (CR) primary bronchial epithelial cells (pBECs) obtained from healthy controls (*n* = 7) and COPD donors (*n* = 7) were assessed for transepithelial electrical resistance, immunofluorescence, cilia activity, ion channel function and expression of cell-type-associated markers at the transcript level. COPD cultures exhibited reduced ciliated area, accompanied by significantly decreased forskolin/IBMX-stimulated CFTR-mediated anion transport and ATP-induced calcium-activated chloride currents compared with healthy-control cultures, despite comparable CFTR mRNA expression. Treatment with CFTR potentiators (VX-770, GLPG1837 and icenticaftor) resulted in modest and highly variable functional responses, with no statistically significant improvement compared with vehicle-treated controls. CFTR function was not significantly associated with cumulative smoking exposure (pack-years) in this cohort, suggesting that smoking burden alone may not account for the observed inter-individual variability in acquired CFTR dysfunction. Collectively, these findings demonstrate abnormalities in ion transport and ciliation in the COPD airway epithelium consistent with an acquired CFTR dysfunction phenotype, but indicate that these abnormalities are not readily reversed by the tested CFTR potentiators. These results highlight differences between genetic and acquired CFTR dysfunction and underscore the need for alternative or combinatorial therapeutic strategies targeting epithelial dysfunction in COPD.

## 1. Introduction

Chronic obstructive pulmonary disease (COPD) is a progressive respiratory disorder characterized by persistent, only partially reversible airflow limitation, chronic airway inflammation, and progressive structural remodeling of the small airways [1]. Approximately two thirds of patients exhibit chronic bronchitis, defined by a persistent productive cough for at least three months over two consecutive years. This phenotype is associated with accelerated decline in lung function, increased exacerbation frequency, impaired quality of life, and increased mortality [2,3]. Pathologically, COPD with chronic bronchitis is characterized by persistent airway inflammation, with increased neutrophils, macrophages, and lymphocytes, alongside mucus hypersecretion, goblet cell hyperplasia, airway remodeling, and microbial colonization [4,5,6,7]. More recently, advanced CT imaging has shown that patients with COPD have airway-occluding mucus plugs that are associated with worse symptoms, impaired lung function and an increased risk of exacerbation [8]. A subset of patients also develop bronchiectasis, reflecting more severe airway structural damage [9].

These pathological features overlap with those observed in cystic fibrosis (CF), a genetic disease caused by mutations in the cystic fibrosis transmembrane conductance regulator (CFTR), an epithelial anion channel that conducts Cl^−^ and HCO_3_^−^. CFTR plays a central role in regulating airway surface liquid (ASL) volume and composition, and its dysfunction leads to impaired mucociliary clearance, mucus stasis, chronic inflammation, and persistent infection, which are central features of chronic airway disease [10]. The development of highly effective CFTR modulators, including potentiators and correctors, has transformed CF care by partially restoring CFTR function and substantially improving pulmonary function, nutritional status, and survival [11,12,13].

Consistent with this, there is increasing recognition that CFTR dysfunction may also contribute to non-CF airway diseases, including COPD. Cigarette smoke, the principal risk factor for COPD, has been shown to induce acquired CFTR dysfunction in individuals without CFTR mutations. Both in vitro and in vivo studies demonstrate that cigarette smoke reduces CFTR-mediated ion transport through multiple mechanisms, including channel internalization, altered gating, and reduced protein stability [14,15,16]. This acquired dysfunction has been implicated in mucus dehydration and defective mucociliary clearance and increased susceptibility to infection in COPD.

These observations have led to the hypothesis that restoring CFTR function could ameliorate key pathological features of COPD. However, while preclinical studies report improvements in ion transport and mucus properties following CFTR activation, clinical outcomes have been inconsistent [17]. Early pilot studies suggested that ivacaftor (VX-770), a CFTR potentiator that enhances channel gating, may augment CFTR activity and improve selected symptoms in COPD [18], but larger trials have shown acceptable safety without significant improvement in lung function [19]. Similarly, icenticaftor, another CFTR potentiator, has shown modest symptomatic benefit without consistent FEV1 improvement [20]. Collectively, these findings suggest that CFTR dysfunction in COPD is mechanistically distinct from CF and may not be fully corrected by current modulators.

A major limitation in the field is that most mechanistic studies on CFTR dysfunction in COPD rely on acute cigarette smoke extract (CSE) exposure models, which do not fully recapitulate the chronic, heterogeneous, and patient-specific nature of the disease. Moreover, CFTR function has not been comprehensively characterized in patient-derived primary bronchial epithelial cells differentiated at the air–liquid interface (ALI), an approach that permits the assessment of donor-derived epithelial function. As a result, it remains unclear whether CFTR dysfunction in the COPD airway epithelium is primarily associated with cumulative smoking exposure or also reflects additional disease-specific factors.

Here, we address this gap using conditionally reprogrammed primary bronchial epithelial cells (CRpBECs) derived from COPD donors and differentiated at the ALI without exogenous smoke exposure. We hypothesized that, if cumulative cigarette smoke were the dominant determinant of acquired CFTR dysfunction, CFTR functional impairment would be associated with smoking burden. Conversely, a lack of association would suggest that additional factors contribute to CFTR dysfunction.

In this study, COPD-derived cultures exhibited impaired epithelial barrier integrity, reduced ciliation, and reduced CFTR- and CaCC-mediated anion transport despite comparable CFTR mRNA expression. Residual CFTR activity was detectable but showed only modest and heterogeneous responses to CFTR potentiators. These findings provide a functional characterization of ion transport abnormalities in the COPD airway epithelium and highlight the limited and variable responsiveness to current CFTR-targeted therapies.

## 2. Results

### 2.1. Donor Characteristics Define a Clinically Relevant COPD Cohort Enriched for a Chronic Bronchitis Phenotype

The clinical characteristics of healthy controls and COPD donors are summarized in Table 1. COPD donors were significantly older than healthy controls (70 ± 7.4 vs. 54 ± 13.8 years, *p* = 0.026) and had a substantial cumulative smoking history (53.3 ± 38.9 pack-years, Supplementary Table S3), whereas controls were non-smokers. Consistent with moderate-to-severe airflow limitation, COPD donors exhibited markedly reduced lung function compared to controls, including FEV1 (% predicted: 44.3 ± 6.8 vs. 90.1 ± 8.3, *p* = 0.0006), FVC (% predicted: 68.6 ± 14.0 vs. 96.6 ± 11.1, *p* = 0.004), and FEV_1_/FVC ratio (51.8 ± 7.5 vs. 73.2 ± 6.4, *p* = 0.001).

Symptom burden, assessed by the COPD Assessment Test (CAT), was elevated in COPD donors (total CAT: 15.9 ± 2.7), with prominent cough (CAT1: 2.43 ± 0.53) and sputum production (CAT2: 2.14 ± 0.89). Based on combined CAT1+CAT2 scores (>2), the majority of COPD donors were consistent with a chronic bronchitis phenotype. None of the COPD patients had bronchiectasis.

Together, these data establish a clinically well-characterized COPD cohort with features relevant to airway mucus dysfunction, providing a robust framework for interpreting epithelial functional phenotypes.

### 2.2. COPD Airway Epithelial Cultures Exhibit Reduced Barrier Function

**Figure 1:**
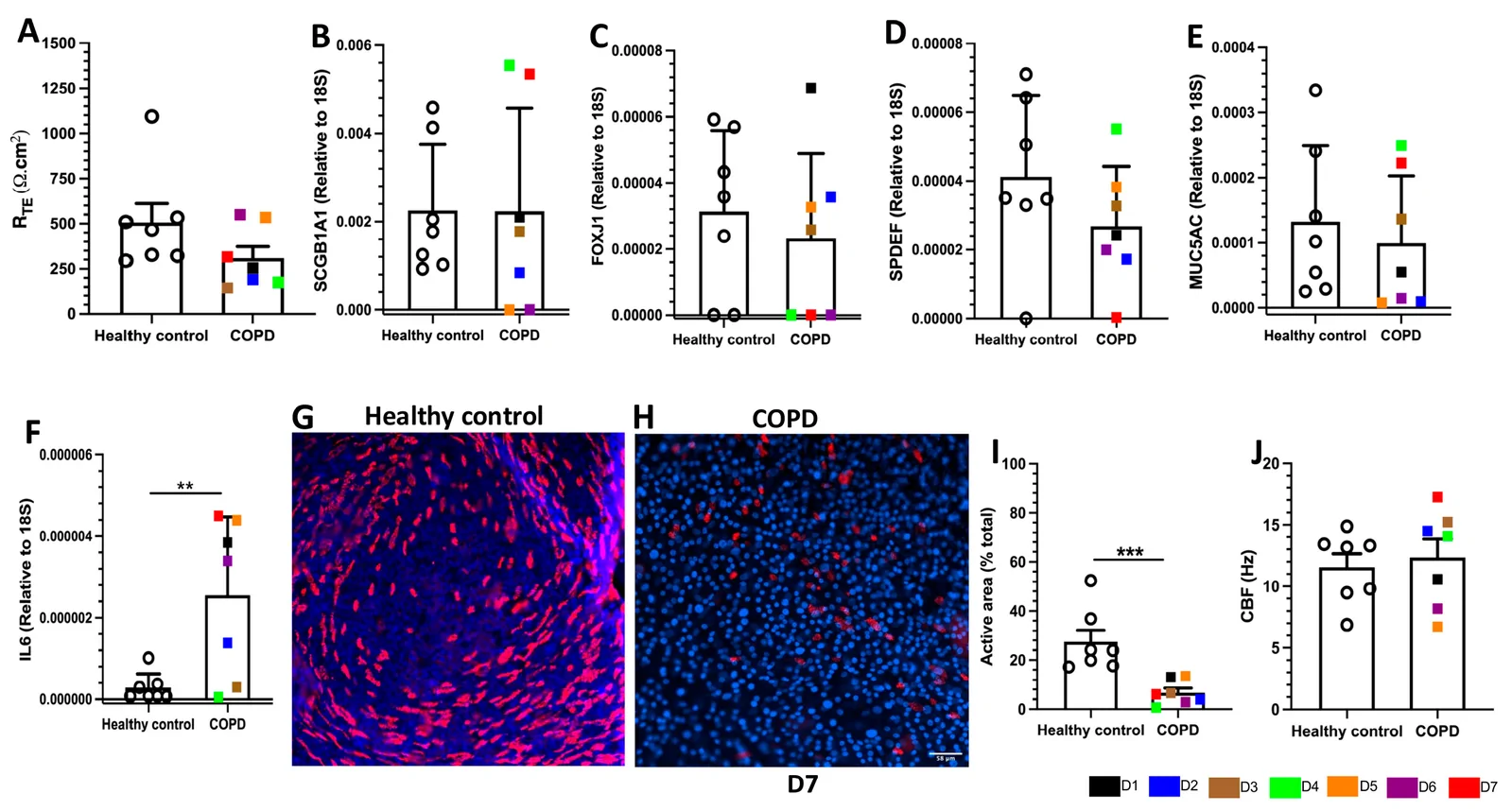
Characterization of epithelial barrier function, epithelial cell type-associated marker expression, and ciliation in healthy control and COPD CRpBEC ALI cultures. (**A**) Trans-epithelial electrical resistance (TEER, RTE) values of healthy controls (*n* = 7) and COPD (*n* = 7) ALI cultures. Each TEER data point represents an average of 10–15 Transwells from each donor. Gene expression of (**B**) SCGB1A1, (**C**) FOXJ1, (**D**) SPDEF, (**E**) MUC5AC and (**F**) IL-6 is reported as [fold change (2^ddct^)], Supplementary Table S1. Representative images of (**G**) healthy controls (Scale bar: 100 μm) and (**H**) COPD (Scale bar: 58 μm) immunofluorescence staining of ciliated cells (anti-acetylated α-tubulin, red) with DAPI (blue). (**I**) Active cilia area and (**J**) cilia beat frequency measurements in healthy controls and COPD ALI cultures. Five different fields of view were sampled per donor. Open circles represent healthy control donors and filled squares with different colors represent different COPD donors. Data presented as mean ± SEM (**A**,**I**,**J**) and mean ± SD (**B**–**F**). Data were analyzed using unpaired *t*-test (**A**,**I**,**J**) or Mann–Whitney U test (**B**–**F**), “*p* < 0.05” was considered significant. COPD donors (D): D1: Black, D2: Blue, D3: Brown, D4: Green, D5: Orange, D6: Purple and D7: Red. ** *p* < 0.01, *** *p* < 0.001.

Primary CRpBECs from healthy controls and COPD donors were differentiated at ALI for 28 days. Epithelial barrier integrity was assessed by TEER and displayed inter-individual variability in both groups. COPD cultures exhibited lower TEER values compared to healthy controls (308.9 ± 63.79 vs. 506.8 ± 104.5 Ω.cm^2^), although this did not reach statistical significance (*p* = 0.137) (Figure 1A). TEER values ranged from 295 to 1094 Ω.cm^2^ for healthy-control cultures and from 143.1 to 549.5 Ω.cm^2^ in COPD cultures.

Despite this reduction, ZO-1 (tight junction protein) localization remained comparable between healthy and COPD cultures (Figure S1A), indicating that tight junction organization was maintained. These findings suggest that although epithelial barrier function was modestly reduced in COPD cultures, gross epithelial architecture remained intact. Given that epithelial barrier integrity and differentiation are closely linked, we next assessed the expression of epithelial cell-type-associated markers.

### 2.3. Reduced Ciliation in COPD CRpBEC ALI Cultures

We then examined whether epithelial differentiation profiles were altered by assessing cell-specific markers using qPCR. No significant difference was observed in the expression of the club cell marker (SCGB1A1) between the COPD and healthy control ALI cultures (Figure 1B). Likewise, the expression of FOXJ1, a marker of ciliogenesis (Figure 1C); SPDEF, a goblet cell marker (Figure 1D); and MUC5AC, a marker of mucus secretory cells (Figure 1E) did not differ significantly between the COPD and healthy control ALI cultures (FOXJ1, *p* = 0.336; SPDEF, *p* = 0.152, MUC5AC, *p* = 0.281).

Notably, expression of the proinflammatory cytokine *IL-6* was significantly increased in COPD cultures compared to controls (*p* = 0.009; Figure 1F), indicating a heightened inflammatory state.

Immunofluorescence analysis further revealed a marked reduction in ciliated cell abundance in COPD cultures. While healthy-control cultures exhibited evenly distributed ciliated cells, COPD cultures showed sparse and clustered ciliation (anti-acetylated tubulin, red, Figure 1G,H and Figure S2A–C). Quantification confirmed an approximately fourfold reduction in active ciliated area, from 27.42 ± 4.85% in healthy controls to 6.65 ± 1.86% in COPD cultures (*p* = 0.0006; Figure 1I).

Despite this reduction in ciliated cell abundance, ciliary beat frequency (CBF) was comparable between groups (COPD: 12.36 ± 1.48 Hz vs. controls: 11.56 ± 1.08 Hz), with substantial inter-individual variability observed in both cohorts (Figure 1J).

Collectively, these findings demonstrate reduced ciliation and increased inflammatory marker expression in COPD-derived airway epithelial cultures. We next evaluated functional epithelial ion transport in these cultures.

### 2.4. COPD ALI Cultures Exhibit Impaired CFTR- and CaCC-Mediated Anion Transport

**Figure 2:**
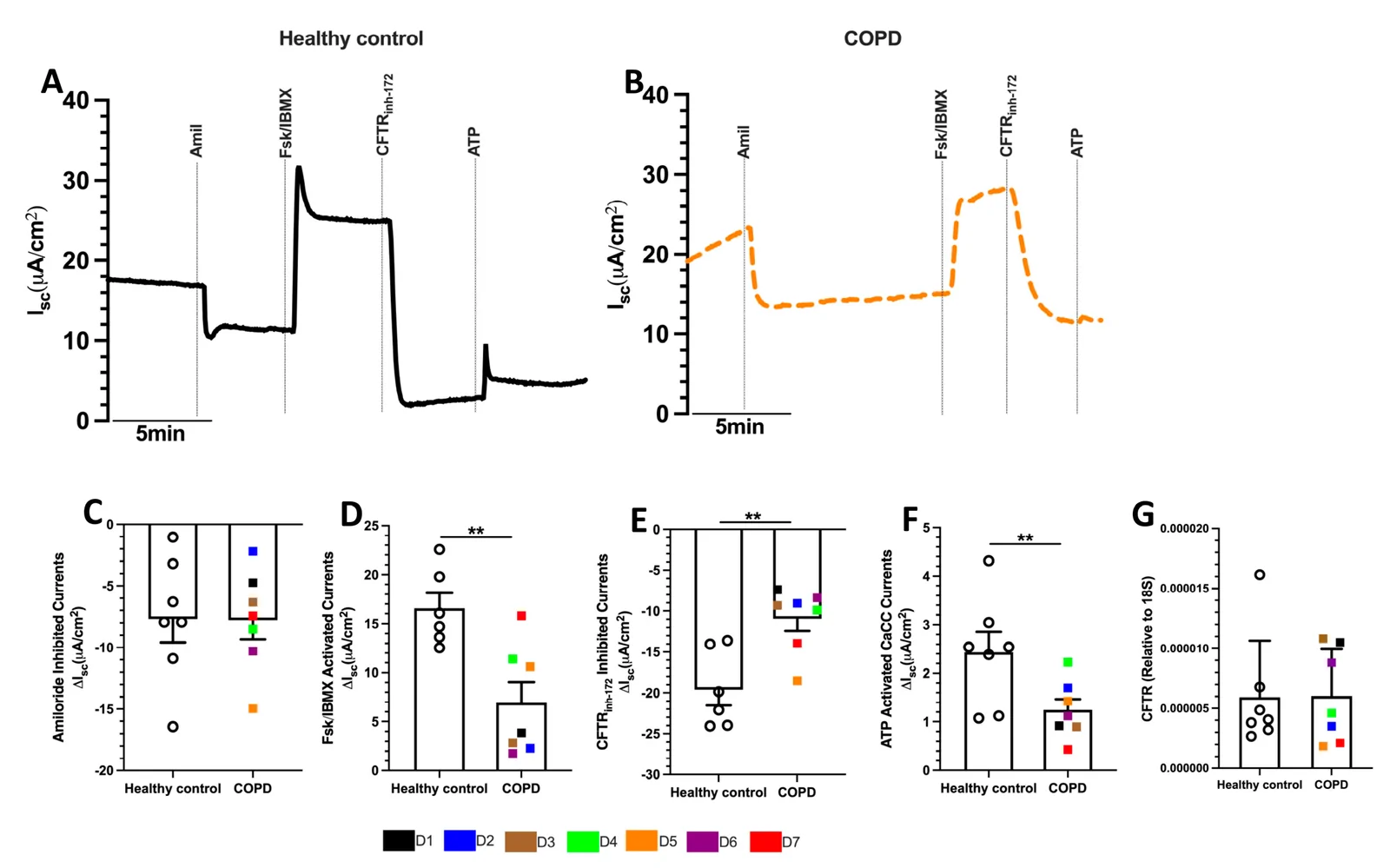
Transepithelial ion transport measurements in healthy controls and COPD CRpBEC ALI cultures. Representative Ussing chamber short circuit current (I_sc_) tracings from (**A**) healthy controls (black line) and (**B**) COPD (dashed orange line), recorded at 37 °C. Mean values of (**C**) amiloride-sensitive ENaC currents, (**D**) forskolin (Fsk) + IBMX activated CFTR currents, (**E**) CFTR_inh-172_ inhibited CFTR currents, (**F**) ATP-activated CaCC currents “*n* = 6” for healthy controls and “*n* = 7” for COPD donors, and (**G**) CFTR mRNA expression levels in healthy controls and COPD cultures. Data presented as mean ± SEM (**C**–**F**) and mean ± SD (**G**). Open circles represent healthy control donors and filled squares with different colors represent different COPD donors. Data were analyzed using unpaired *t*-test, “*p* < 0.05” was considered significant. ** *p* < 0.01.

Transepithelial ion transport in healthy control and COPD ALI cultures was assessed by measuring amiloride-inhibited sodium channel (ENaC) currents, forskolin (Fsk)/IBMX stimulated and CFTR_inh-172_-inhibited CFTR currents and ATP-induced calcium-activated chloride channel (CaCC) currents (Figure 2A,B). Some heterogeneity was observed in ion channel measurements in both healthy controls and COPD cultures (Figure 2C–F).

Amiloride-sensitive ENaC currents did not differ significantly between healthy control and COPD cultures (ΔI_sc-Amiloride_ −7.67 ± 1.90 vs. −7.78 ± 1.55 µA/cm^2^, respectively; *p* = 0.966; Figure 2A–C), indicating that basal sodium absorption was preserved in COPD-derived airway epithelium.

CFTR-mediated Cl^−^ secretion was assessed using cAMP agonist Fsk and phosphodiesterase inhibitor IBMX, followed by CFTR-specific inhibitor CFTR_inh-172_. COPD cultures exhibited significantly impaired Fsk/IBMX-induced currents, approximately 2.5 times lower than healthy-control cultures (ΔI_sc-Fsk/IBMX_ 6.64 ± 2.10 vs. 16.55 ± 1.58 µA/cm^2^, *p* = 0.008; Figure 2A,B,D). A similar finding was observed in CFTR-inhibited currents, whereby COPD cultures displayed significantly lower CFTR_inh-172_-inhibited currents compared to healthy-control cultures (ΔI_sc-CFTR-inh-172_ −10.93 ± 1.48 vs. −19.60 ± 1.94 µA/cm^2^, *p* = 0.008; Figure 2A,B,E).

In addition to CFTR dysfunction, ATP-induced calcium-activated chloride channel (CaCC) currents were also significantly reduced in COPD cultures, approximately twofold lower than those in healthy-control cultures (ΔI_sc-ATP_ 1.24 ± 0.22 vs. 2.43 ± 0.42 µA/cm^2^, *p* = 0.025; Figure 2A,B,F), indicating that epithelial anion transport defects in COPD are not restricted to CFTR but also involve alternative chloride secretory pathways.

Despite these functional abnormalities, CFTR mRNA expression was comparable between healthy control and COPD cultures (Figure 2G). These findings suggest that transcriptional downregulation alone is unlikely to account for the observed reduction in CFTR-mediated anion transport. However, CFTR protein expression, maturation, glycosylation, trafficking, and membrane localization were not assessed; therefore, the mechanisms underlying the observed functional impairment cannot be determined from the present study.

Together, these results demonstrate coordinated impairment in epithelial chloride secretion in the COPD airway epithelium, affecting both CFTR-dependent and alternative anion transport pathways.

### 2.5. CFTR Potentiators Produce Modest and Heterogeneous Functional Responses in COPD Airway Epithelium

Given the presence of residual CFTR activity despite functional impairment, we next investigated whether pharmacological potentiation could improve CFTR function in COPD cultures. CFTR potentiators VX-770, GLPG1837, and icenticaftor were tested in selected COPD donors representing relatively high (D4 and D7, Supplementary Table S2) and low (D5 and D6, Supplementary Table S2) baseline CFTR activity.

**Figure 3:**
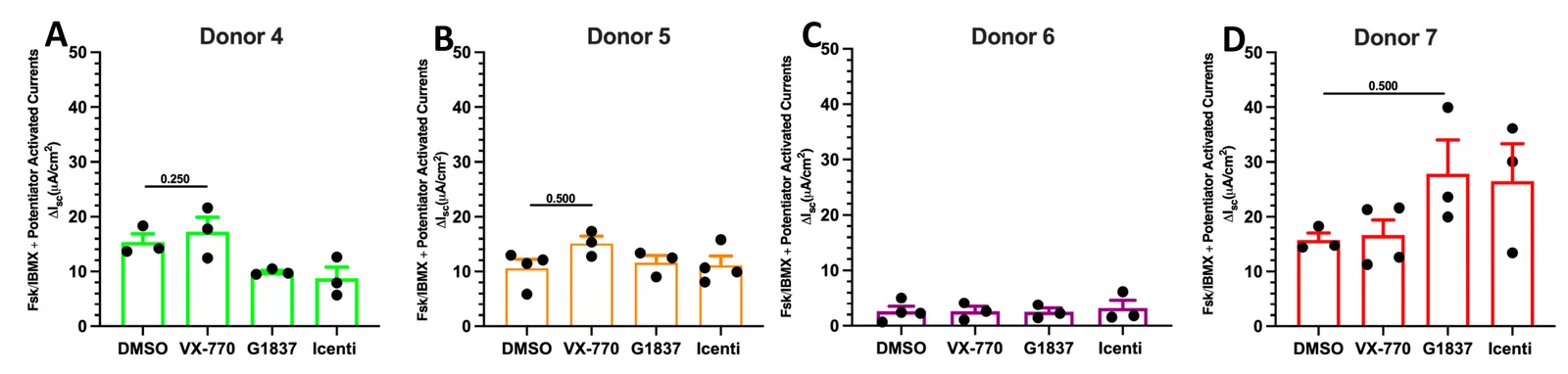
CFTR functional response of COPD CRpBEC ALI cultures following treatment with CFTR potentiators VX-770, GLPG1837 and icenticaftor. (**A**–**D**) represent bar-graph summaries of the mean values of Forskolin/IBMX + respective potentiator activated currents. CFTR potentiator response was not tested in COPD donors 1 to 3 (baseline CFTR activity). Each closed circle represents a single Transwell insert, with 3–4 Transwell inserts analyzed per donor per treatment. Data are presented as mean ± SEM. Paired Wilcoxon test was used to determine statistical significance, “*p* < 0.05” was considered significant.

All potentiators produced variable, donor-dependent increases in CFTR-mediated currents. Treatment with VX-770 in COPD donors 4, 5, and 7 induced a modest improvement in CFTR restoration compared to DMSO (vehicle), ranging from 0.87 to 4.54 µA/cm^2^. However, this difference did not reach statistical significance (Figure 3A,B,D and Supplementary Figure S3A–E,G). Treatment with potentiator GLPG1837 in COPD donors 5 and 7 increased Fsk/IBMX currents by 1.00 and 12.03 µA/cm^2^ respectively when compared to DMSO (Figure 3B,D and Supplementary Figure S3B–E,G). Treatment with icenticaftor in donors 5, 6, and 7 increased CFTR currents compared to DMSO, ranging between 0.52 and 10.71 µA/cm^2^ (Figure 3B–D and Supplementary Figure S3B–G).

Overall, these findings indicate that while individual donors displayed evidence of improved CFTR function in response to one or more potentiators, the effects were modest, highly variable, and did not reach statistical significance across all samples regardless of baseline CFTR activity. These findings are consistent with residual CFTR-mediated functional activity but do not establish CFTR protein abundance or localization at the epithelial surface.

### 2.6. CFTR-Mediated Anion Transport Is Not Significantly Associated with Cumulative Smoking Exposure

**Figure 4:**
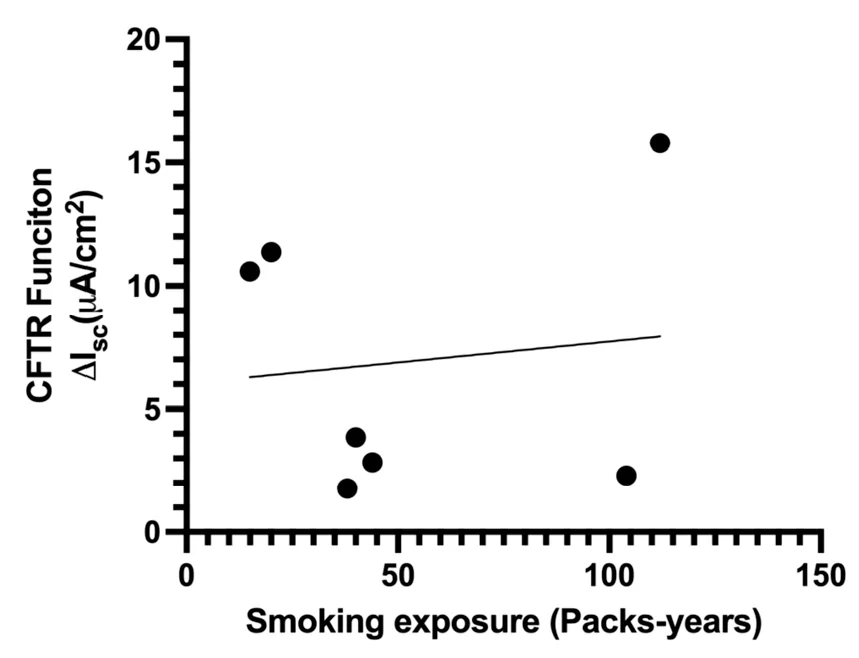
Relationship between smoking exposure and CFTR-mediated ion transport in primary bronchial epithelial cultures. Scatter plot showing the relationship between cumulative smoking exposure (pack-years) and CFTR-dependent short-circuit current (ΔIsc, µA/cm^2^) across individual donors (*n* = 7). Each point represents a single donor. Statistical analysis (Pearson’s correlation, two-tailed) confirms no significant association (r = 0.12, 95% CI −0.70 to 0.80, *p* = 0.80).

We next assessed whether cumulative cigarette exposure (pack-years) was associated with CFTR-mediated anion transport in COPD donors. No significant correlation was observed between pack-years and CFTR function (Pearson r = 0.12, 95% CI −0.70 to 0.80, *p* = 0.80, Figure 4), indicating that smoking burden alone does not predict the extent of CFTR dysfunction. Notably, donors with high smoking exposure exhibited divergent CFTR activity, with some individuals demonstrating markedly impaired function (e.g., D2), while others retained relatively high CFTR activity (e.g., D7). These findings highlight substantial inter-donor variability and suggest that factors beyond smoking exposure contribute to CFTR dysfunction in COPD.

## 3. Discussion

COPD is characterized by airway mucus obstruction, chronic inflammation, and recurrent infection—features that overlap with CF and have led to increasing recognition of acquired CFTR dysfunction in COPD. While this has generated interest in repurposing CFTR-targeted therapies, most experimental models rely on exogenous CSE, which incompletely recapitulates disease complexity. Here, using CRpBECs differentiated at the ALI, we demonstrate abnormalities in epithelial barrier function, ciliation, and ion transport, including reduced CFTR- and CaCC-mediated anion transport, in the absence of acute smoke exposure.

### 3.1. Intrinsic Epithelial Dysfunction in COPD

Following ALI differentiation, COPD-derived cultures exhibited reduced barrier integrity (lower TEER) and a significantly reduced ciliated area compared with healthy-control cultures. Expression of epithelial cell-type-associated markers FOXJ1, SPDEF, and MUC5AC did not differ significantly between COPD and healthy-control cultures. Together with the reduced ciliated area, these findings are consistent with previous reports describing epithelial remodeling in COPD [21,22,23,24,25,26,27]. Recent studies have shown that CFTR is predominantly expressed in ionocytes and secretory epithelial cells rather than multiciliated cells [28,29,30]. Therefore, the reduced ciliated area observed in COPD cultures is unlikely to directly account for the impaired CFTR-mediated anion transport. However, secretory cell and ionocyte populations were not specifically quantified in the present study, and their potential contribution to reduced CFTR function remains to be determined.

### 3.2. Relationship of CFTR Dysfunction to CFTR mRNA Expression and Cumulative Smoking Exposure

A central finding of this study is that CFTR-mediated anion transport is markedly reduced in COPD cultures despite comparable CFTR mRNA expression, indicating that transcriptional downregulation alone is unlikely to account for the observed functional impairment. This is consistent with prior human and animal studies [17,31]. Previous studies have identified several mechanisms that may contribute to acquired CFTR dysfunction, including altered channel gating, internalization, and reduced protein stability [14,15,16]. Inflammatory processes may also contribute, as neutrophil-derived proteases have been shown to degrade CFTR in the COPD airway [32].

Importantly, we observed no significant correlation between cumulative smoking exposure (pack-years) and CFTR-mediated anion transport in this cohort, with individuals of similar smoking histories exhibiting markedly different levels of CFTR activity. Although the small cohort limits conclusions regarding the relationship between smoking burden and CFTR dysfunction, these findings suggest that cumulative smoking exposure alone may not account for the observed inter-individual variability in CFTR function. Consistent with this, acquired CFTR dysfunction is not universal in COPD, with previous studies reporting dysfunction in only a subset of individuals, including up to ~40% of smokers [33,34]. Together, these observations suggest that additional factors, including inflammatory and epithelial processes, may contribute to the heterogeneity of acquired CFTR dysfunction in COPD.

### 3.3. Divergent Regulation of Epithelial Ion Channels in COPD

Beyond CFTR, we identified a reduction in calcium-activated chloride channel (CaCC) activity in COPD cultures, which may reflect impaired calcium signaling or the altered expression of regulators such as ORAI3 (ORAI—Calcium release-activated calcium modulator 3), as previously reported [35]. In contrast, ENaC activity was not significantly altered in COPD cultures, diverging from observations in CSE-based models [36,37,38]. This discrepancy highlights differences between acute exposure systems and patient-derived epithelial models and underscores the value of primary cell systems in capturing disease-relevant physiology.

### 3.4. Residual CFTR Activity Provides a Rationale for Therapeutic Potentiation

Despite reduced function, COPD cultures retained substantial residual CFTR-mediated activity (~50–60% of healthy controls), providing a rationale for pharmacological potentiation. CFTR potentiators increase channel open probability and/or conductance [39], with the potential to improve airway hydration, mucociliary clearance, and host defense [15]. Consistent with this, indirect CFTR activation has been implicated in the clinical efficacy of agents such as roflumilast [40], suggesting that CFTR modulation may contribute to therapeutic benefit in COPD.

### 3.5. Limited and Heterogeneous Responses to CFTR Modulators

Despite this rationale, CFTR potentiators (VX-770, GLPG1837, and icenticaftor) produced modest, highly variable, and non-significant improvements in CFTR function across COPD donors. These heterogeneous responses provide a mechanistic explanation for the inconsistent outcomes observed in clinical trials, where ivacaftor and icenticaftor have shown limited or delayed efficacy [19,20].

Several factors likely contribute to the limited responsiveness to CFTR potentiators observed in COPD cultures. First, acquired CFTR dysfunction in COPD may arise through multiple mechanisms, including oxidative modification, altered channel gating, and proteolytic degradation of CFTR, as reported in previous studies. Such mechanisms may differentially influence responsiveness to potentiator-based rescue. Second, reduced apical membrane CFTR abundance, as previously reported in COPD and cigarette smoke-exposed airway epithelium, could limit the amount of functional CFTR available for pharmacological potentiation. Third, pharmacokinetic factors and suboptimal drug exposure in vivo may further influence therapeutic efficacy in patients with COPD.

### 3.6. Mechanistic Basis of Variability and Therapeutic Resistance

The marked inter-donor variability observed in this study highlights the heterogeneity of COPD at the molecular level. Cigarette smoke-derived reactive species can induce oxidative and covalent modifications of CFTR, potentially altering channel conformation in ways that are not rescued by potentiators. In parallel, smoke-induced internalization and epithelial remodeling may reduce functional channel availability. These effects may be compounded by distinct disease endotypes that differentially influence CFTR dysfunction and drug responsiveness. Notably, tobacco smoke exposure itself may antagonize modulator efficacy, as suggested in CF populations [41]. Collectively, these observations suggest that CFTR dysfunction in COPD is mechanistically heterogeneous and may not be amenable to a uniform therapeutic strategy.

### 3.7. Therapeutic Implications and Future Directions

Taken together, our data suggest that CFTR potentiators alone may be insufficient to restore acquired CFTR dysfunction in COPD. More effective approaches may require combination strategies incorporating CFTR correctors and/or amplifiers, or emerging modalities such as CFTR mRNA delivery. Mechanistically informed patient stratification will be important for identifying the individuals most likely to benefit from CFTR-targeted interventions.

### 3.8. Limitations

This study has several limitations. The relatively small donor cohort limits statistical power and may not fully capture the biological heterogeneity of COPD, particularly with respect to CFTR function and potentiator responsiveness. CFTR genotyping was not performed, and CFTR protein abundance, processing, trafficking, and membrane localization were not assessed; therefore, the molecular basis of the observed functional impairment cannot be established. CFTR potentiators were evaluated at a single screening concentration and were not systematically assessed in healthy control ALI cultures, limiting concentration–response analysis and direct comparison of pharmacological responsiveness between healthy and COPD-derived epithelium. Although minimally expanded P1 cells were used, the potential effects of in vitro expansion and conditional reprogramming on epithelial phenotype cannot be completely excluded. Furthermore, epithelial cell composition was not comprehensively quantified by cell-type-specific immunofluorescence, flow cytometry, or single-cell analysis; therefore, bulk qPCR measurements of cell-type-associated markers should not be interpreted as direct measures of cellular abundance. In particular, pulmonary ionocytes were not directly quantified, and future studies incorporating ionocyte-specific immunofluorescence or single-cell approaches will be required to determine whether ionocyte abundance contributes to differences in CFTR function between donors. Finally, the ALI model does not reproduce ongoing cigarette smoke exposure, the inflammatory milieu, or other environmental influences present in vivo.

## 4. Materials and Methods

### 4.1. Human pBEC Procurement and Processing

The study was performed in accordance with approvals from the Hunter New England Area Health Service Ethics Committee (Ref. No. 05/08/10/3.09; approval date: 1 January 2006) and the University of Newcastle (Newcastle, NSW, Australia) Human Research Ethics Committee (H-163-1205; approval date: 14 December 2005). Human pBECs were obtained from healthy controls and individuals with COPD (*n* = 7 donors per group) by endobronchial brushing during fiber-optic bronchoscopy [42]. COPD donors had stage II or III as defined by the Global Initiative for Obstructive Lung Disease (GOLD) guidelines [43]. Written informed consent was obtained from all study participants before sample collection. Patient-derived pBECs were expanded in standard BEGM media (BEGM; Lonza, Basel, Switzerland) and cryopreserved for later use.

### 4.2. NIH/3T3 Feeder Cell Culture and Irradiation

NIH/3T3 mouse embryonic fibroblast cells were cultured in DMEM (Sigma D5796, St. Louis, MO, USA) supplemented with 10% FBS and 1% (*v*/*v*) penicillin/streptomycin (Life Technologies, Scoresby, Australia). At 80–90% confluency, cells were trypsinized, resuspended in fresh culture media and exposed to 30 Gy γ-irradiation (RS 2000 X-Ray irradiator, RAD SOURCE, Buford, GA, USA). Irradiated NIH/3T3 cells were seeded into collagen I coated flasks (PureCol; Advanced Biomatrix 5005, Carlsbad, CA, USA) at a 1:1 ratio with pBECs, as previously described.

### 4.3. Conditional Reprogramming (CR) Cell Expansion Culture/Co-Culture

Cryopreserved pBECs were revived and expanded in BEGM media. To establish CRpBECs, passage 1 (P1) pBECs were co-cultured with an equal number of irradiated NIH/3T3 feeder cells in collagen I-coated flasks containing F-media supplemented with the ROCK inhibitor Y-27632, as described previously [44]. After cultures reached 50% confluence, cells were gradually weaned off Y-27632, and no Y-27632 was added to the culture media after 85% confluency [44]. Conditionally reprogrammed pBECs were separated from the irradiated 3T3 feeder cells by differential trypsinization. Briefly, cultures were exposed to trypsin/EDTA (Lonza CC-5034) for 2 min at 37 °C to detach the feeder cells while the epithelial cells remained adherent. After removal of the feeder cells, the remaining pBECs were detached by continued trypsinization for 5–7 min at 37 °C, neutralized with trypsin neutralizing solution (Lonza CC-5024), collected, and used for ALI differentiation. Cell number and viability were obtained using the trypan-blue method.

### 4.4. Air-Liquid Interface (ALI) Cultures

Upon reaching confluence, P1 CRpBECs were harvested and seeded onto collagen I coated 24-well Transwell membranes (Sigma CLS3470) at a density of 1.5 × 10^5^ cells/insert and cultured in bronchial epithelial base medium and Dulbecco’s modified eagle medium (BEBM:DMEM, 50:50) differentiation medium, as described previously [44]. To minimize culture-induced changes in epithelial phenotype, all ALI cultures were established using minimally expanded P1 cells. Apical media was removed after cells reached confluence (3–5 days) to establish ALI conditions. Media in the basal compartment was changed every second day for 28 days. The apical compartment was washed once weekly at 37 °C with phosphate-buffered saline (PBS, no Ca^2+^ and Mg^2+^) to remove excess mucus.

### 4.5. Transepithelial Electrical Resistance (TEER) Measurements

TEER measurements were performed on days 7, 14, 21 and 28 of ALI cultures using an EVOM2 Voltohmmeter and STX2 electrodes (World Precision Instruments, Sarasota, FL, USA). TEER values (expressed as Ω·cm^2^) were calculated by subtracting the resistance of a blank reference insert (without cells) and correcting for the insert surface area (0.33 cm^2^).

### 4.6. Transepithelial Ion Transport Assay

Differentiated CRpBEC ALI cultures (28–30 days) were mounted in circulating Ussing chambers (Physiologic Instruments VCC MC8 multichannel voltage/current clamp). Short-circuit current (I_sc_) was measured under voltage-clamp conditions, with at least three Transwells analyzed per donor per condition. For I_sc_ recordings, cells were bathed in 5 mL of 37 °C Krebs-bicarbonate-Ringer containing (mM): 115 NaCl, 25 NaHCO_3_, 2.4 K_2_HPO_4_, 0.4 KH_2_PO_4_, 1.2 CaCl_2_, 1.2 MgCl_2_ and 10 glucose, (pH 7.4), continuously gassed with 95% O_2_-5% CO_2_. After 15 min stable baseline I_sc_ recording, cells were sequentially treated with pharmacological compounds: (1) 100 µM amiloride (apical), (2) 5 µM VX-770 or 5 µM GLPG1837 or 5 µM icenticaftor (apical), each tested separately as a standardized screening concentration to assess acute CFTR potentiation; (3) 10 µM forskolin and 100 µM IBMX (apical and basolateral), (4) 30 µM CFTR_inh-172_ (apical) and (5) 100 µM ATP (apical). Data were analyzed using Acquire and Analyze software (v2.3, Physiologic Instruments, Venice, FL, USA).

### 4.7. Cilia Beating Frequency and Active Area Measurements

Live imaging of ciliary beating in CRpBEC ALI cultures (frequency range, 3–20 Hz) was performed using a Nikon eclipse Ti2 microscope (Nikon, Tokyo, Japan) connected to a high-speed digital video recorder and Video Savant 4.0 software. Images were captured at 300 frames per second (fps), with a minimum of 512 frames acquired per recording. Five fields of view were captured at random for each donor per insert. Data analysis for median cilia beat frequency was performed using the CiliaFA plugin [45] together with free open-source Fiji-ImageJ software (v1.53, Image J, Bethesda, MD, USA). Ciliary active area was calculated using the in-built ‘Stack difference’ analysis in Fiji-Image J software by highlighting areas of ciliary motion for each 512-frame file. Thresholding was applied identically to all projected images and active areas measured. Results from each sample represent the mean of five fields of view.

### 4.8. Whole-Mount Immunolabelling and Fluorescent Microscopy

CRpBEC ALI cultures were fixed in 4% paraformaldehyde for 15 min followed by storage in PBS containing 50 mM Glycine at 4 °C, as previously described [46]. Membranes were permeabilized with 0.1% *v*/*v* Triton-X 100, blocked with 10% *v*/*v* goat serum in PBS and incubated overnight at 4 °C with antibodies against acetylated tubulin (T7451, Sigma) and ZO-1 (33-9100, Invitrogen, Waltham, MA, USA). After washing with PBS, membranes were incubated with anti-mouse Alexa Fluor 594 secondary antibody (8890, Cell signaling technology, Danvers, MA, USA). Membranes were mounted using Fluoromount-G containing DAPI (00-4959-52, Invitrogen, USA). A minimum of three images were captured per donor at random using a Nikon Eclipse DS-Qi2 microscope fitted with a CoolLED pE-300 illumination system. Images were processed using ImageJ software (National Institute of Health, Bethesda, MD, USA).

### 4.9. RNA Extraction, cDNA Synthesis, and Quantitative PCR

Total RNA was extracted from CRpBEC ALI cultures lysed in RLT buffer containing β-mercaptoethanol (QIAGEN) using RNeasy Mini Kits (QIAGEN, Hilden, Germany), according to the manufacturer’s instructions. RNA quality and quantity were measured using a NanoDrop 2000 spectrophotometer (Thermo Scientific, Scoresby, Australia). RNA (200 ng) was reverse-transcribed to cDNA using a High-Capacity cDNA Reverse Transcription Kit (Thermo Scientific). qPCR was performed using a QuantStudio 6 as per manufacturer’s instructions using TaqMan gene expression assays (Thermo Fisher Scientific, Australia). Expression was normalized to the ribosomal RNA (18S) housekeeping gene (Table S1). Relative gene expression was calculated using the 2^−ΔΔCt^ method (where Ct is the threshold cycle), as described previously [47].

### 4.10. Statistical Analysis

Data are represented as dot plots with mean ± standard error of the mean (SEM), except for qPCR data, which are represented as mean ± standard deviation (SD). Unpaired *t*-test, Mann–Whitney U test, and paired Wilcoxon tests were used as appropriate. The correlation between smoking exposure (pack-years) and CFTR function (ΔIsc) was assessed using Pearson’s correlation coefficient (two-tailed), with 95% confidence intervals calculated using Fisher’s z-transformation. Statistical analyses were performed using GraphPad Prism software (v9.3.1, San Diego, CA, USA). A *p* value < 0.05 was considered statistically significant.

## 5. Conclusions

In summary, COPD airway epithelial cultures exhibit abnormalities in epithelial barrier function, ciliation, and ion transport, including acquired CFTR dysfunction and reduced CaCC-mediated anion transport. Although residual CFTR-mediated activity was detected, pharmacological potentiation produced limited and heterogeneous functional improvement. These findings suggest that CFTR dysfunction in COPD is heterogeneous and not solely associated with cumulative smoking exposure, which may help explain the variable efficacy of CFTR modulators reported in COPD. Further mechanistic and patient stratification studies will be required to identify the individuals most likely to benefit from CFTR-targeted therapeutic approaches.

## Figures and Tables

**Table 1:** Clinical characteristics of healthy controls and COPD donors of primary bronchial epithelial cells.

Clinical Characteristics	Healthy Controls	COPD	Difference, *p* Values
Number, *n*	7	7	-
Age, yr (SD)	54 (13.80)	70 (7.40)	0.026
Male, *n* (%)	3 (42)	2 (33)	-
BMI (SD)	29.76 (7.90)	29.50 (6.85)	0.904
Cumulative smoking, pack-years, (SD)	0	53.29 (38.92)	-
FEV1, % predicted (SD)	90.14 (8.27)	44.29 (6.75)	0.0006
FVC, % predicted (SD)	96.57 (11.12)	68.57 (14.01)	0.004
(FEV1/FVC) % (SD)	73.15 (6.37)	51.83 (7.54)	0.001
CAT 1 score (cough) (SD)	0	2.43 (0.53)	-
CAT 2 score (sputum production) (SD)	0	2.14 (0.89)	-
CAT total (SD)	0	15.86 (2.67)	-
Antibiotics ^#^ (SD)	0	2.14 (1.34)	-
OCS ^#^ (SD)	0	1.85 (1.57)	-

Definition of abbreviations: yr: year, SD: standard deviation, FEV1: forced expiratory volume in 1 s, FVC: forced vital capacity, BMI: body mass index, CAT: COPD assessment test, OCS: oral corticosteroids, ^#^: number of courses in the previous 12 months.

## Data Availability

The original contributions presented in this study are included in the article/Supplementary Material. Further inquiries can be directed to the corresponding author.

## Supplementary Materials

The following supporting information can be downloaded at: https://www.mdpi.com/article/10.3390/ijms27198803/s1.
