# Supplementary material for: Assessment of CFTR Dysfunction and Responsiveness to CFTR Modulators in COPD Bronchial Air–Liquid Interface Cultures
Source: Int J Mol Sci. 2026 Oct 1;27(19):8803. doi: 10.3390/ijms27198803 (PMC13664796; doi:10.3390/ijms27198803)
Supplement: Supplementary file 1 [file ijms-27-08803-s001.zip › ijms-4418708-supplementary.pdf]

Supplementary Tables:

Table S1. Genes assessed by PCR

Gene	TaqMan Assay ID	Dye-Probe	Supplier	Catalogue number
FOXJ1	Hs00201755_m1	FAM-MGB	ThermoFisher Scientific	4331182
SPDEF	Hs0017942_m1	FAM-MGB	ThermoFisher Scientific	4331182
SCGB1A1	Hs00171092_m1	FAM-MGB	ThermoFisher Scientific	4331182
MUC5AC	Hs01365616_m1	FAM-MGB	ThermoFisher Scientific	4331182
IL-6	Hs00174131_m1	FAM-MGB	ThermoFisher Scientific	4331182
18s	N/A	VIC-MGB	ThermoFisher Scientific	4319413E

Table S2: Response to CFTR potentiators in COPD donors

Donor	DMSO ΔI_{sc} (μA/cm^2) DMSO + Fsk/IBMX activated currents	VX-770 ΔI_{sc} (μA/cm^2) VX-770 + Fsk/IBMX activated currents	G1837 ΔI_{sc} (μA/cm^2) G1837+ Fsk/IBMX activated currents	Icenticaftor ΔI_{sc} (μA/cm^2) Icenti+ Fsk/IBMX activated currents
D4	15.39 ± 1.48	17.25 ± 2.66[a]	9.88 ± 0.30	8.85 ± 2.02
D5	10.60 ± 1.59	15.14 ± 1.33[b]	11.60 ± 1.31	11.12 ± 1.66
D6	2.59 ± 0.90	2.60 ± 0.88	2.49 ± 0.69	3.16 ± 1.49
D7	15.80 ± 1.24	16.67 ± 2.76	27.83 ± 6.15[c]	26.51 ± 6.79

***D**: Donor, **Fsk**: Forskolin, **IBMX**: 3-Isobutyl-1-methylxanthine, **G1837**: GLPG1837, **Icenti:** Icenticaftor

a = DMSO vs VX-770 + Fsk/IBMX activated current, $p = 0.500$
b = DMSO vs VX-770 + Fsk/IBMX activated current, $p = 0.250$
c = DMSO vs G1837 + Fsk/IBMX activated current, $p = 0.25$

Table S3: COPD donors cigarette packs per year

Donor	Color code	Packs - years	ΔI_{sc} (μA/cm^2) CFTR currents	Gender
D1	black	40	3.86 ± 0.72	Female
D2	blue	104	2.30 ± 1.10	Female
D3	brown	44	2.84 ± 0.29	Female
D4	green	20	11.38 ± 2.54	Female
D5	orange	15	10.60 ± 1.59	Male
D6	purple	38	1.77 ± 0.54	Male
D7	red	112	15.8 ± 1.24	Female

Supplementary figure S1

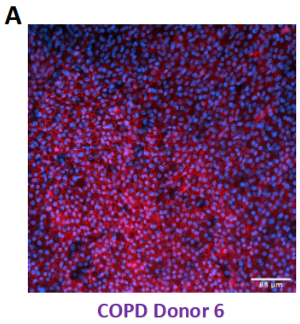

Supplementary Figure S1: ZO-1 staining in COPD cultures

Supplementary figure S2

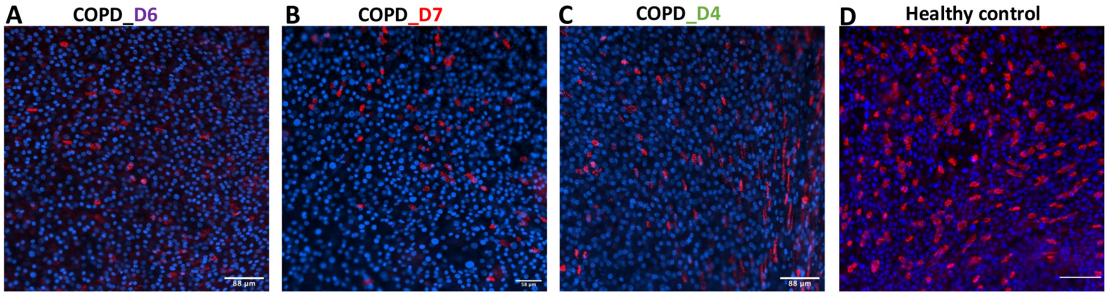

Supplementary Figure S2: Immunofluorescence staining of ciliated cells (anti-acetylated α-tubulin, red) with DAPI (blue) A-C COPD donors and D healthy control

Supplementary figure S3

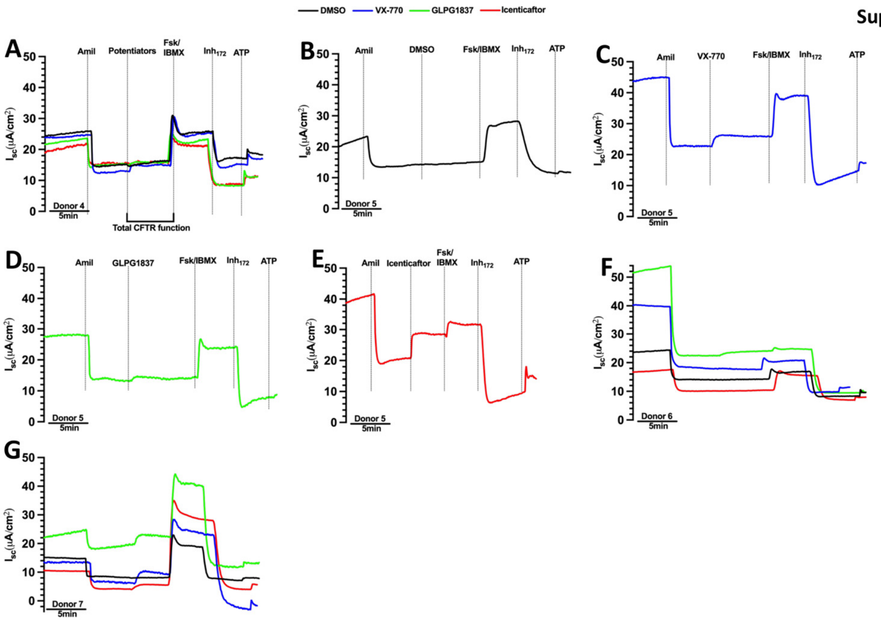

Supplementary Figure S3: Representative Ussing chamber short circuit current (Isc) tracings from Donor 4 (A), Donor 5 (B), Donor 6 (C) and Donor 7 (D).
